# Herb-Drug Pharmacokinetic Interaction of a Traditional Chinese Medicine Jia-Wei-Xiao-Yao-San with 5-Fluorouracil in the Blood and Brain of Rat Using Microdialysis

**DOI:** 10.1155/2015/729679

**Published:** 2015-03-10

**Authors:** Meng-Hsuan Chiang, Li-Wen Chang, Ju-Wen Wang, Lie-Chwen Lin, Tung-Hu Tsai

**Affiliations:** ^1^Institute of Traditional Medicine, National Yang-Ming University, Taipei 112, Taiwan; ^2^National Research Institute of Chinese Medicine, Ministry of Health and Welfare, Taipei 112, Taiwan; ^3^Graduate Institute of Acupuncture Science, China Medical University, Taichung 404, Taiwan; ^4^School of Pharmacy, College of Pharmacy, Kaohsiung Medical University, Kaohsiung 807, Taiwan; ^5^Department of Education and Research, Taipei City Hospital, Taipei 103, Taiwan

## Abstract

According to a survey from the National Health Insurance Research Database (NHIRD), Jia-Wei-Xiao-Yao-San (JWXYS) is the most popular Chinese medicine for cancer patients in Taiwan. 5-Fluorouracil (5-FU) is a general anticancer drug for the chemotherapy. To investigate the herb-drug interaction of JWXYS on pharmacokinetics of 5-FU, a microdialysis technique coupled with a high-performance liquid chromatography system was used to monitor 5-FU in rat blood and brain. Rats were divided into four parallel groups, one of which was treated with 5-FU (100 mg/kg, i.v.) alone and the remaining three groups were pretreated with a different dose of JWXYS (600, 1200, or 2400 mg/kg/day for 5 consecutive days) followed by a combination with 5-FU. This study demonstrates that 5-FU with JWXYS (600 mg/kg/day or 1200 mg/kg/day) has no significant effect on the pharmacokinetics of 5-FU in the blood and brain. However, JWXYS (2400 mg/kg/day) coadministered with 5-FU extends the elimination half-life and increases the volume of distribution of 5-FU in the blood. The elimination half-life of 5-FU in the brain for the pretreatment group with 2400 mg/kg/day of JWXYS is significantly longer than that for the group treated with 5-FU alone and also reduces the clearance. This study provides practical dosage information for clinical practice and proves the safety of 5-FU coadministered with JWXYS.

## 1. Introduction

5-Fluorouracil (5-FU) was discovered by the research group of Dr. Heidelberger in 1957 [1]; up to date, it still remains a widely prescribed agent for the treatment of solid tumors. It has been proven that 5-FU can pass through the blood–brain barrier (BBB) by simple diffusion [2, 3]; however its concentration is not sufficient to be an effective dose [4, 5]. The 5-FU may cause “chemobrain,” which refers to cognitive alterations that involve deficits in executive and motor function and verbal memory. To explore the 5-FU penetration from blood to the brain, the concentrations of 5-FU in the brain should be monitored. In clinical application, 5-FU is usually combined with other anticancer drugs. It may potentially cause short-term or long-term interaction [4, 6, 7].

Currently, many cancer patients seek alternative therapies that reduce the side effects that are associated with other cancer therapies. Traditional Chinese medicine (TCM) and herbal medicine are complementary and alternative medicines (CAM) that have been used for thousands of years especially in Asia, and they have become increasingly popular in the West [8]. Of the many types of CAM, herbal supplements and vitamins are the most popular among patients with cancer. Cancer patients also use herbal supplements in combination with conventional treatments for several reasons, such as an improved quality of life, alleviation of the side effects of chemotherapy, strengthening the immune system, and slowing the progression of cancer [9, 10]. Because of the high frequency with which anticancer drugs are coadministered with herbal medicine, the herb-drug interaction has become increasingly important.

According to the National Health Insurance Research Database (NHIRD) from 1999 to 2008 in Taiwan, the herb medicine formula, Jia-Wei-Xiao-Yao-San (JWXYS; meaning augmented rambling powder), was the most frequently prescribed formula for patients with gastric cancer and the second most commonly prescribed formula for those with breast cancer [11]. JWXYS is also a widely used herbal formula for the treatment of agitation, functional dyspepsia, and insomnia and it is also used as a mood stabilizer [12–14]. These symptoms are often observed in cancer patients.

The 5-FU and JWXYS are also commonly coadministered for the cancer patients. However, the pharmacokinetic interaction of JWXYS on 5-FU is still unclear. In order to figure out that the coadministration of 5-FU and JWXYS induces herb-drug interaction, multiple microdialysis probes were implanted into the jugular vein and the brain striatum, for respective sampling of the blood and brain samples. The experimental rats are divided into four parallel groups: one being treated with 5-FU (100 mg/kg, i.v.) alone and the other three individual groups being pretreated with different doses of JWXYS (600, 1200, or 2400 mg/kg/day for 5 consecutive days), followed by a combination with 5-FU (100 mg/kg, i.v.). Furthermore, for quality control of the herbal extract, the six main bioactive components, that is, paeoniflorin, saikosaponin A, saikosaponin D, glycyrrhizic acid, z-ligustilide, and ferulic acid, of JWXYS extract were detected by liquid chromatography-tandem mass spectrometry (LC-MS/MS) system.

## 2. Materials and Methods

### 2.1. Chemicals and Reagents

5-FU, *α*-chloralose, and urethane were provided by Sigma-Aldrich Chemicals (St. Louis, MO, USA). Crushed JWXYS herbs were purchased from a Chinese traditional herbal medicine store in Taipei and prepared in the National Research Institute of Chinese Medicine, Taipei, Taiwan. Citric acid, sodium citrate, dextrose, sodium chloride (NaCl), potassium chloride (KCl), calcium chloride (CaCl_2_), potassium dihydrogen phosphate (KH_2_PO_4_), sodium hydroxide (NaOH), ammonium acetate (NH_4_OAC), and methanol of HPLC grade were purchased from E. Merck (Darmstadt, Germany). Deionized water from Millipore (Milford, MA, USA) was used for all aqueous solutions in the experiment.

### 2.2. The Herbal Preparation of Jia-Wei-Xiao-Yao-San

The herbal composition and plant parts used in this study followed the Chinese medicinal prescription of the Ministry of Health and Welfare in Taiwan (2013) and Chinese Pharmacopoeia (2010). JWXYS was prepared by mixing 10 crude herbs and plant parts used in this study were as follows:* Radix Angelicae Sinensis* (used part: root, Chinese herbal name: Dang-Gui),* Rhizoma Atractylodis Macrocephalae* (used part: rhizome, Chinese herbal name: Bai-Zhu),* Radix Bupleuri* (used part: root, Chinese herbal name: Chai-Hu),* Poriae Cocos* (used part: sclerotium, Chinese herbal name: Fu-Ling),* Radix Paeoniae Alba* (used part: root, Chinese herbal name: Bai-Shao),* Radix Glycyrrhizae Uralensis* (used parts: root and rhizome, Chinese herbal name: Zhi-Gan-Cao),* Cortex Moutan Radicis* (used part: root bark, Chinese herbal name: Mu-Dan-Pi),* Fructus Gardeniae* (used part: ripe fruit, Chinese herbal name: Zhi-Zi),* Rhizoma Zingiberis Recens* (used part: rhizome, Chinese herbal name: Wei-Jiang), and* Herba Menthae* (used parts: stem and leaf, Chinese herbal name: Bo-He). In line with the Chinese medicinal prescription of the Ministry of Health and Welfare in Taiwan, the ratio of JWXYS ingredients was 4 : 4 : 4 : 4 : 4 : 2 : 2.5 : 2.5 : 4 : 2 (w/w, resp.) on a dry weight basis. The herbal mixture was soaked in deionized water (20 times the volume of the mixture) for herbal preparation. After boiling for 1 hour, the herbal mixture was filtered from decoction and the water extract was concentrated by heating the mixture at 70°C for 2.5 h. The concentrated water extract of JWXYS was finally freeze-dried to a powder and stored at −20°C until used. The yield of the extract was 17.44%.

### 2.3. LC-MS/MS for Herbal Analysis

The LC-20AD UPLC system (Shimadzu Co., Kyoto, Japan) consisted of a LCMS-8030 (Shimadzu Co., Kyoto, Japan) triple quadrupole mass spectrometer equipped with LC-20AD pump (Shimadzu Co., Kyoto, Japan), CTO-20A column oven (Shimadzu Co., Kyoto, Japan), DGU-20A3 online degasser (Shimadzu Co., Kyoto, Japan), and SIL-20ACXR autosampler (Shimadzu Co., Kyoto, Japan). Furthermore, the tandem quadrupole mass spectrometer equipped with electrospray ionization (ESI) turbo ion interface was used with the following parameters: interface voltage (kV): 4.5; DL temperature (°C): 250; heat block temperature (°C): 400; nebulizing gas flow (L/min): 3; and drying gas flow (L/min): 15 and nitrogen was used in all cases.

A C18 column (ACQUITY BEH, 100 mm × 2.1 mm i.d., particle size 1.7 mm, Waters, Ireland) was used for UPLC separation. The mobile phase consisted of (A) methanol with 0.1% formic acid and (B) 5 mM NH_4_OAC. The gradient elution program of mobile phase was as follows: 0-1 min: 25–70% A; 1–3.5 min: 70–90% A; 3.5–7.5 min: 90-90% A; 7.5–8.5 min: 90–25% A; and 8.5–13 min: 25-25% A, v/v. The flow rate for the mobile phase was set at 0.2 mL/min and the analytical volume was 5 *μ*L of JWXYS extract.

### 2.4. Experimental Animals

The protocol was reviewed and approved by the Institutional Animal Care and Use Committee (IACUC, approval number 1021001) and by the Institutional Animal Experimentation Committee of National Yang-Ming University, Taipei, Taiwan. Male Sprague-Dawley rats (220–280 g) were supplied by the Laboratory Animal Center at National Yang-Ming University (Taipei, Taiwan). The animals were housed in a pathogen-free environment and maintained on a 12 h light-dark cycle. They had free access to food (laboratory rodent diet 5P14, PMI Feeds, Richmond, IN, USA) and water* ad libitum*. All animal experiments followed the National Yang-Ming University guidelines and procedures for the care of laboratory animals. The study was conducted over a 5-day period and the rats were randomly divided into 4 treatment groups: group (1), 5-FU (100 mg/kg, i.v.) administered alone, group (2), a daily dose of JWXYS (600 mg/kg/day, p.o.) pretreated for 5 consecutive days and on the 5th day + 5-FU (100 mg/kg, i.v.), group (3), a double dose of JWXYS (1200 mg/kg/day, p.o.) pretreated for 5 consecutive days and on the 5th day + 5-FU (100 mg/kg, i.v.), and group (4), high dose of JWXYS (2400 mg/kg/day, p.o.) pretreated for 5 consecutive days and on the 5th day + 5-FU (100 mg/kg, i.v.).

### 2.5. Drug Administration

**Figure 1:**
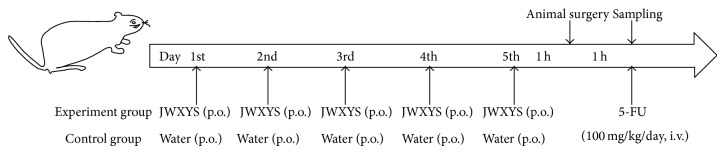
The study design for the drug administration. Rats were divided into four parallel groups, one of which was treated with 5-FU (100 mg/kg, i.v.) alone and the remaining three groups were pretreated with a different dose of JWXYS (600, 1200, or 2400 mg/kg/day for 5 consecutive days) followed by a combination with 5-FU.

In this study, the dose was calculated using a formula for dose translation from humans to rats. This formula uses the body surface area normalization method to convert the dose from humans to rats [15]. In clinical therapy, the dosage of 5-FU was 600 mg/m^2^, for the initial treatment and maintenance therapy. Using the formula, the human equivalent dosage of 5-FU in rats is 100 mg/kg. A previous study revealed that, comparing the dose-normalized area under the curve (AUC) at different intravenous doses of 5-FU in rats, the dose-normalized AUC after the administration of 100 mg/kg is greater than 50 mg/kg, or 10 mg/kg [16], so 100 mg/kg was chosen as a suitable 5-FU dose for rats to determine the 5-FU pharmacokinetic parameters. The extract powder of JWXYS was dissolved in deionized water at a concentration of 100 mg/mL, for oral administration by gavages to the rats. A daily dose of JWXYS extract for humans is 5.8 g once a day for adults. This equates to 600 mg/kg/day for rats and this dosage is called a daily dose in this study. However, the dose of JWXYS depends on the symptoms, so a double dose (1200 mg/kg/day) and a high dose (2400 mg/kg/day) of JWXYS were also used to determine the herb-drug interaction in detail. Rats in group 1 were initially anesthetized using an anesthetic mixture (10 mL/kg, i.p.) of urethane (1 g/kg) and *α*-chloralose (0.01 g/mL) and were then given 5-FU (100 mg/kg, i.v.) alone through the femoral vein. Groups 2–4 were pretreated with different doses of JWXYS for 5 consecutive days and on the 5th day, 1 h after pretreatment with JWXYS, the rats were anesthetized with the anesthetic mixture (10 mL/kg, i.p.) to perform surgery and another 1 h was taken to balance the microdialysis device after the animal experiment concluded. 5-FU (100 mg/kg, i.v.) was finally injected into the femoral vein. The administration protocol was shown in Figure 1.

### 2.6. Microdialysis Experiment

In this study, concentrations of 5-FU in blood and the brain should be monitored simultaneously. Microdialysis allows continuous monitoring of drug concentrations at various tissue sites in a single animal, avoids the problems with intra-animal variability, reduces the number of animals used, and provides AUC data directly to calculate the ratio of drug penetration from blood to the brain [17]. The microdialysis system consisted of a microinjection pump (CMA/100, CMA, Stockholm, Sweden), a microfraction collector (CMA/140), and microdialysis probes. The microdialysis probes for blood and the brain were made of silica capillary with a concentric design and the tips were covered by a dialysis membrane (molecular weight cutoff of 13,000 Da, Spectrum, Laguna Hills, CA, USA). The active lengths were 1 cm and 5 mm, for blood and brain, respectively [17]. Both types of probes for blood and the brain were made in the authors' laboratory similarly to previous studies [17, 18]. When the rats had been anesthetized, a polyethylene tube (PE-50; Clay Adams, NJ, USA) was cannulated in the femoral vein for 5-FU administration. A microdialysis probe for blood was implanted into the jugular vein, toward the right atrium, and used anticoagulant dextrose (ACD) solution (citric acid 3.5 mM; sodium citrate 7.5 mM; dextrose 13.6 mM) as a perfusate (the fluid entering the microdialysis probe). A microdialysis probe for the brain was also positioned within the striatum zone and the perfusate for the brain was Ringer's solution (consisting of NaCl 8.6 g; KCl 0.3 g; CaCl_2_ 0.33 g in 1000 mL H_2_O; pH 7.0). The flow rate for the perfusate was set at 2.0 *μ*L/min, using an autoinjection pump. After the surgery for tube cannulation and implanting the microdialysis probes, one hour of postsurgical stabilization was required. 5-FU (100 mg/kg, i.v.) was then injected into the femoral vein. The dialysates (the fluid flowing out of the microdialysis probe) were collected by an autocollector every 15 min for 3 h and stored at −20°C for analysis. Compared with traditional sampling methods, microdialysis is a technique for protein unbound drug sampling due to the molecular weight limit of probe membrane and offers a very clean dialysate. Hence the dialysates required no further clean-up procedure or extraction before being injected directly into the HPLC-UV system [17]. All of the samples were determined using a HPLC-UV system (Shimadzu Co., Kyoto, Japan).

### 2.7. Instrumentations and HPLC-UV Conditions

The HPLC system consisted of chromatographic pumps (LC-20AT; Shimadzu Co., Kyoto, Japan), an autosampler (SIL-20AC; Shimadzu Co., Kyoto, Japan), and an UV-Vis detector (SPD-M20A; Shimadzu Co., Kyoto, Japan). All analytical samples were separated using a reverse-phase C18 column (Purospher STAR, 250 mm × 4 mm i.d.; particle size 5 *μ*m, Merck, Darmstadt, Germany). The mobile phase for HPLC analysis consists of two solvent compositions: 10 mM potassium phosphate and methanol (99 : 1, v/v). The pH of 10 mM potassium phosphate was adjusted to pH 6 for the blood samples, using sodium hydroxide, but there was no such adjustment for the brain samples. The flow rate for the mobile phase was set at 0.8 mL/min. The temperature in the autosampler was set at 8°C, the analytical volume was 20 *μ*L of each sample, the UV-Vis detector scanned from 200 to 500 nm, and the chromatographic profiles were monitored at 266 nm for 5-FU.

### 2.8. Method Validation

The standard stock solution of 5-FU (1 mg/mL) was prepared in methanol and working standard solutions were diluted using 50% (v/v) methanol. Calibration curves were generated by spiking different concentrations of the working solutions in blank rat dialysates for the blood and brain. The calibration curves range from 0.5 to 100 *μ*g/mL for the blood and from 0.1 to 10 *μ*g/mL for the brain. The linearity of the assay was checked using the coefficient of determination (*r* ^2^) for the calibration curve, which should be greater than 0.995. The limit of detection (LOD) was estimated as the concentration that yields a signal to noise of 3 and the lower limit of quantification (LLOQ) was defined as the lowest concentration of the linear regression. The precision and accuracy of this analytical method were verified by preparing six identical calibration curves on the same day (intraday) and on six successive days (interday). The accuracy (bias, %) is calculated using the nominal concentration (*C*_nom⁡_) and the mean value for the observed concentrations (*C*_obs_), as follows:

(1) $$\begin{array}{c} \text{Bias}\left(\%\right)=\left[\frac{\left(C_{\text{obs}}-C_{\mathrm{nom}}\right)}{C_{\mathrm{nom}}}\right]\times 100. \end{array}$$

The precision (RSD, %) is calculated using the standard deviation and the observed concentration (*C*_obs_), as follows:

(2) $$\begin{array}{c} \text{RSD}\left(\%\right)=\left[\frac{\text{standard}\;\text{deviation}\left(\text{SD}\right)}{C_{\text{obs}}}\right]\times 100. \end{array}$$

The mean values for the accuracy and the precision must be within 15% of the actual value, except at the LLOQ, where it must not exceed the value by more than 20%. The method validation for this study was performed according to the guidance given by the US Food and Drug Administration [19].

### 2.9. *In Vivo* Recovery of Microdialysis Probes

5-Fluorouracil is a water-soluble and small molecule drug [20]; therefore, it can be dissolved in perfusate. The microdialysis probes recovery of 5-FU was estimated by an* in vivo* relative loss method. The* in vivo* relative loss method determines the drug dialyzed from the perfusate into the dialysate to measure the exchange efficiency of the dialysis membrane in relation to the drug [21, 22]. Three different concentrations of 5-FU were diluted in ACD and Ringer solution for the blood and brain microdialysis probes (*n* = 3 for each type). The brain and blood microdialysis probes were then placed under the same conditions as described for the sampling of free-form 5-FU in the blood and the brain. The samples and perfusates that contained 5-FU were then analyzed simultaneously. The concentration of 5-FU in the perfusate (*C*_perf_) and that in the collected dialysate (*C*_dial_) was determined by HPLC-UV. The relative* in vivo* recovery (*R*, %) for 5-FU across the dialysis membrane is calculated as *R* = [(*C*_perf_ − *C*_dial_)/*C*_perf_] × 100%.

The actual 5-FU concentration (*C*_act_) in target organ is calculated as *C*_act_ = *C*_obs_/*R*. *C*_obs_ is the observed concentration of 5-FU in the microdialysis samples.

### 2.10. Data Analysis

Each individual set of data was used to calculate the pharmacokinetic parameters, using the pharmacokinetic program, WinNonlin Standard Edition Version 1.1 (Scientific Consulting, Apex, NC, USA). The pharmacokinetic parameters include the maximum concentration of 5-FU (*C*_max⁡_), the area under the concentration versus time curve (AUC), the elimination half-life (*t*_1/2_), the clearance (CL), and the apparent volume of distribution (Vd). The ratio of 5-FU penetration from blood to the brain is calculated as follows: (AUC_brain_/AUC_blood_) × 100(%). AUC_brain_ is the AUC for the brain for each group and AUC_blood_ is the AUC for the blood in the 5-FU only treatment group.

The statistics are determined using an analysis of variance in the SPSS 18.0 program (SPSS Inc., Chicago, USA). All data are presented as mean ± standard error of mean (S.E.M.). Student's *t*-test or one-way ANOVA was used to compare the differences between groups and a *P* value <0.05 was considered as the level of significance.

## 3. Result and Discussion

### 3.1. LC-MS/MS for Herbal Analysis

**Figure 2:**
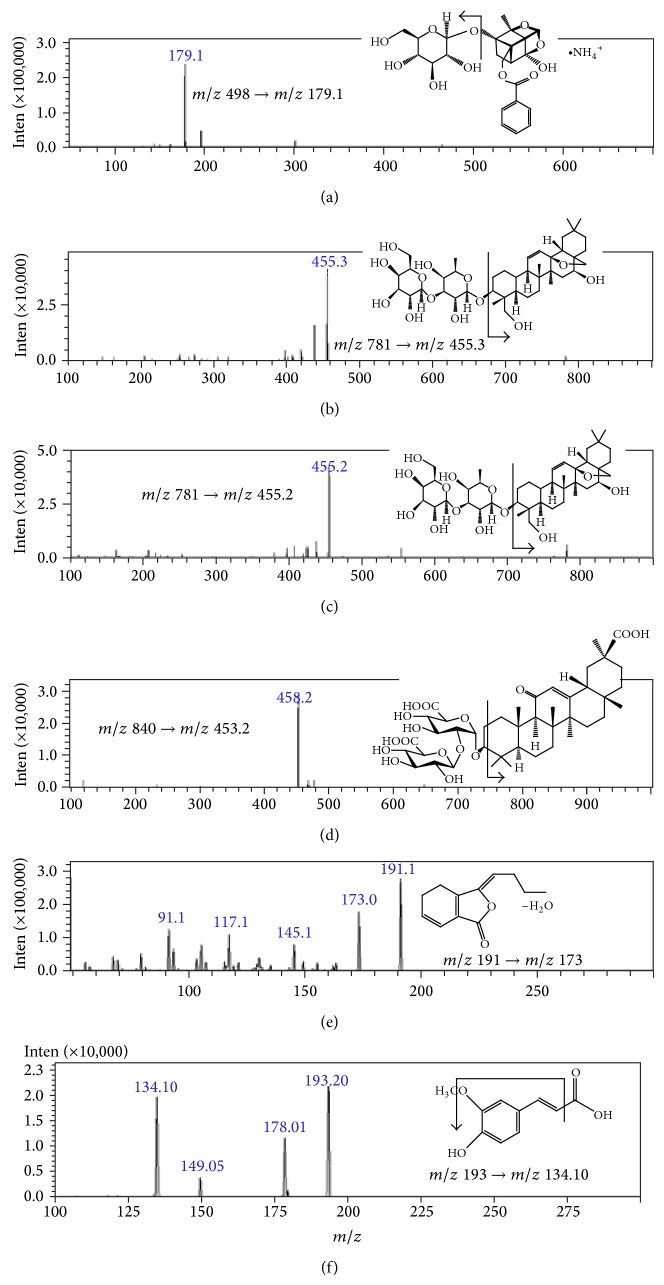
The mass spectra of precursor and product ions for six marker compounds in JWXYS extract: (a) paeoniflorin, (b) saikosaponin A, (c) saikosaponin D, (d) glycyrrhizic acid, (e) z-ligustilide, and (f) ferulic acid.

Qualification of six biomarker compounds in JWXYS extract was performed by UPLC-MS/MS. The following precursor → product ion transitions were used to identify the herbal ingredients: *m*/*z* 498.00 → 179.10 for paeoniflorin; *m*/*z* 781.40 → 455.35 for saikosaponin A; *m*/*z* 781.40 → 455.20 for saikosaponin D; *m*/*z* 840.30 → 453.20 for glycyrrhizic acid; *m*/*z* 191.00 → 173.00 for z-ligustilide; and *m*/*z* 193.00 → 134.10 for ferulic acid (Figure 2).

### 3.2. Optimization of HPLC-UV Conditions

**Figure 3:**
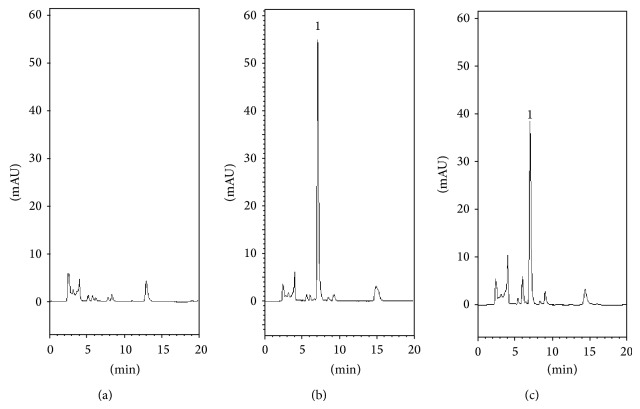
HPLC chromatograms of (a) blank blood dialysate; (b) standard 5-FU (10 *μ*g/mL) spiked with blank blood dialysate; and (c) blood sample containing 5-FU (7.1 *μ*g/mL) collected at 60 min after 5-FU (100 mg/kg, i.v.) administration alone. Peak 1: 5-FU and retention time of 5-FU was 7.1 min.

**Figure 4:**
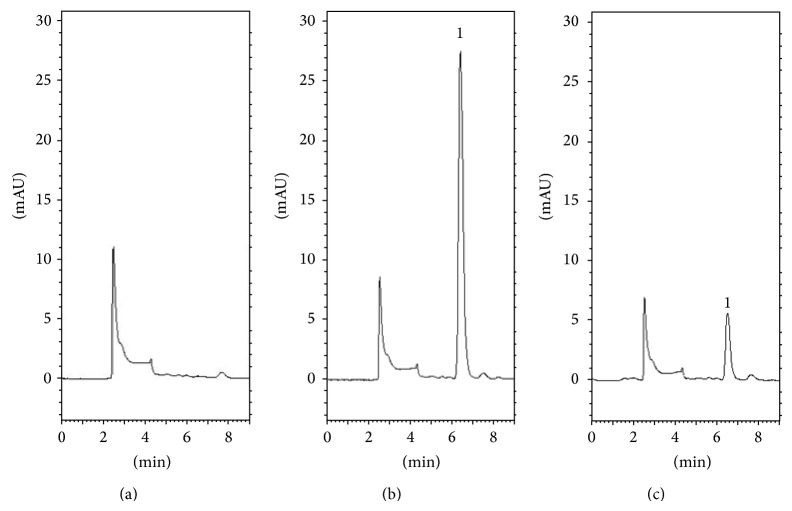
HPLC chromatograms of (a) blank brain dialysate; (b) standard 5-FU (5 *μ*g/mL) spiked with blank brain dialysate; and (c) brain sample containing 5-FU (0.9 *μ*g/mL) collected at 15 min after 5-FU (100 mg/kg, i.v.) administration alone. Peak 1: 5-FU and retention time of 5-FU was 6.1 min.

To optimize the analytical system, the pH value of the buffer system must be determined. Previous studies showed that a reverse-phase C18 column with the mobile phase of 1% methanol and 99% of 10 mM KH_2_PO_4_ (v/v) produces acceptable separation of 5-FU in plasma [23, 24]. However, 5-FU plasma samples were collected by withdrawing from the jugular vein, not microdialysis system. In order to improve the sensitivity and accuracy of the determination, the analytical methods used to ascertain the pH value of the mobile phase for blood and the brain were different. Because of the different compositions of the dialysates for blood and the brain, various pH values for the buffer solution 10 mM KH_2_PO_4_ were used. The optimization process showed that pH 6.0 10 mM KH_2_PO_4_, adjusted using 0.1 M NaOH, was suitable for blood sample analysis and without adjusting the pH value of the buffer solution, it was suitable for analysis of the brain samples. HPLC chromatograms for 5-FU in rat blood and brain were shown in Figures 3 and 4, respectively. The retention time for 5-FU was 7.1 min in blood samples and 6.7 min in brain samples. In this study, microdialysis was used for sampling because the analyte was obtained from the endogenous matrix. Compared to the blank dialysate and analyte spiked dialysates, there were no observed peaks that interfere with the analytes within the retention time of 5-FU and the result shows good selectivity for the analytical method used in this study.

### 3.3. Method Validation for Linearity, Precision, and Accuracy

To validate the analytical system, the limit of detection (LOD) was determined at a signal-to-noise ratio (*S*/*N*) of 3, for the same chromatographic conditions. In this study, LOD was 0.05 *μ*g/mL for blood dialysate and 0.01 *μ*g/mL for brain dialysate. In order to conform to the various concentrations of 5-FU in the blood and the brain, the calibration curves for different ranges were examined. The examination shows that the calibration curves have good linearity (*r* ^2^ > 0.999) over the range of 0.5−100 *μ*g/mL for blood dialysate and 0.1−10 *μ*g/mL for brain dialysate. In addition, the mean values for the regression equations for 5-FU are *y* = 81491*x* + 6489.8 (*r* ^2^ = 0.999) and *y* = 84354*x* + 450.23 (*r* ^2^ = 0.999) in rat blood and brain dialysates, respectively. The LLOQ values for the analytical method are 0.5 *μ*g/mL for the blood sample and 0.1 *μ*g/mL for the brain sample. The determination of the interday and intraday precision and accuracy for the analytical method was conducted by spiking blank dialysates with concentrations in the range of the calibration curves. The interday and intraday precision and accuracy for 5-FU in blood and brain were summarized in Tables 1 and 2. All results for the examination are within the acceptable criteria of ±15%, except at LLOQ, where the value is within 20%. These results show that the method was reliable and valid for the analysis of 5-FU in the dialysates of blood and brain.

### 3.4. *In Vivo* Recovery of Microdialysis Probes

To correct the dialysate concentration and extracellular concentration,* in vivo* recovery was used to evaluate the recovery of the microdialysis probes. In microdialysis experiment, 5-FU in dialysate was collected by gradient diffusion from the target tissue, through the semimembrane to the perfusate, so not all of 5-FU in the target tissue could be collected. Therefore, it was important to estimate the recovery for the microdialysis probe.* In vivo* recovery of the microdialysis probes was conducted by using an* in vivo* relative loss method. In this estimation, low, middle, and high concentrations within the range of the calibration curves are used to spike with perfusate. In addition, a previous study shows that the recovery of probes is related to the resistance caused by sampling tissue and the active length of probe membrane [25]. The resistance in brain tissue is higher than blood and the active length of dialysis membrane in the brain (5 mm) is shorter than blood (1 cm); therefore, the recovery of brain probes is lower than blood probes. The results show that the recovery is independent of the 5-FU concentrations for both blood and brain probes and it is also consistent with the results for a previous study [21]. The microdialysis device is shown to be stable and reliable within the range of the calibration curves. The average recovery of 5-FU in different concentrations is 50.35 ± 2.93% in the blood probe and 9.32 ± 1.32% in the brain probe (Table 3). These data are used to convert the observed concentration of 5-FU in dialysate into the actual concentration in the target organ.

### 3.5. Pharmacokinetics of 5-FU in Blood

**Figure 5:**
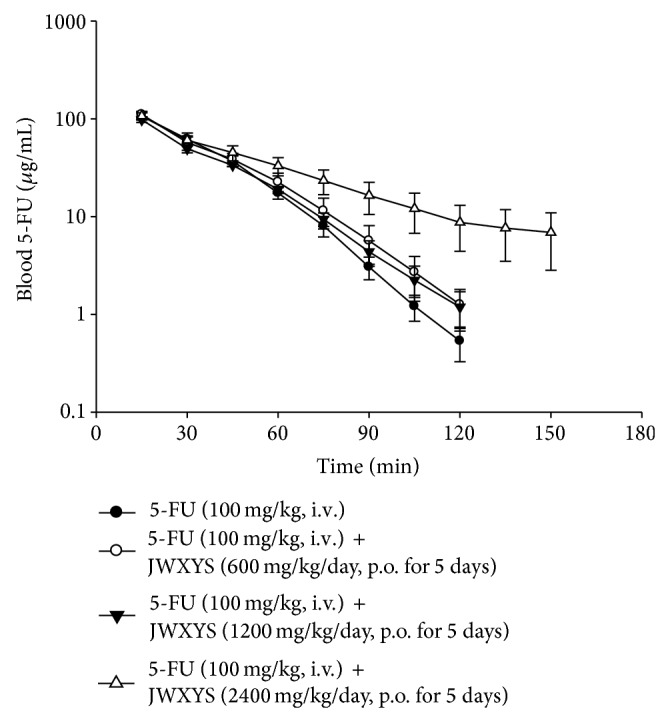
Concentration-time curve of 5-FU in rat blood dialysate after 5-FU administration (100 mg/kg, iv) alone and pretreated with a different dose of JWXYS (600, 1200, or 2400 mg/kg/day for 5 consecutive days). Data were expressed as means ± S.E.M. (*n* = 6).

The blood concentration versus time profile for 5-FU for the groups treated with 5-FU alone (100 mg/kg, i.v.) and pretreated with various doses of JWXYS is shown in Figure 5. The result shows that the concentration of 5-FU is not significantly different for the group pretreated with a daily dose of JWXYS (600 mg/kg/day) and a double dose JWXYS (1200 mg/kg/day) for five consecutive days. However, the profile shows that pretreatment with a high dose of JWXYS (2400 mg/kg/day) can prolong the residence time of 5-FU in blood. The results show that *C*_max⁡_ for 5-FU in blood is 107 ± 10 *μ*g/mL, 111 ± 9 *μ*g/mL, 98 ± 6 *μ*g/mL, and 107 ± 8 *μ*g/mL following intravenous administration of the four groups, and the AUC for 5-FU in blood is 5020 ± 443 min *μ*g/mL, 5516 ± 486 min *μ*g/mL, 4801 ± 244 min *μ*g/mL, and 6159 ± 898 min *μ*g/mL for each group. The CL values are 20.8 ± 2.0 mL/min per kg, 18.7 ± 1.4 mL/min per kg, 21.0 ± 1.0 mL/min per kg, and 17.6 ± 2.6 mL/min per kg, following intravenous administration for the four groups (Table 4). After coadministration of 5-FU with JWXYS, there are no significant differences in the AUC, *C*_max⁡_, and CL values between each group and there is also no difference between the *t*_1/2_ and Vd values for the 5-FU alone (11.0 ± 0.6 min and 448 ± 51 mL/kg) and 5-FU coadministered with a daily or a double dose of JWXYS (13.1 ± 1.6 min and 406 ± 45 mL/kg; 13.3 ± 1.6 min and 495 ± 76 mL/kg).

Comparing the group treated with 5-FU alone and the group treated with a high dose of JWXYS (2400 mg/kg/day) pretreatment, the *t*_1/2_ value for the high dose JWXYS pretreatment group in blood is 25.6 ± 5.2 min, which is higher than the *t*_1/2_ value for 5-FU alone (11.0 ± 0.6 min), and the Vd value for the high dose of JWXYS pretreatment group (575 ± 41 mL/kg) is also significantly increased, compared with those for the group treated with 5-FU alone (448 ± 51 mL/kg) (Table 4). The AUC value increases when 5-FU is coadministered with a high dose of JWXYS, but the difference is not significant. In summary, this study demonstrates that only a high dose of JWXYS (2400 mg/kg/day) coadministered with 5-FU leads to an increase in the elimination half-life and the apparent volume of distribution of 5-FU and may lead to an increase of the AUC in the blood.

### 3.6. Pharmacokinetics of 5-FU in the Brain

**Figure 6:**
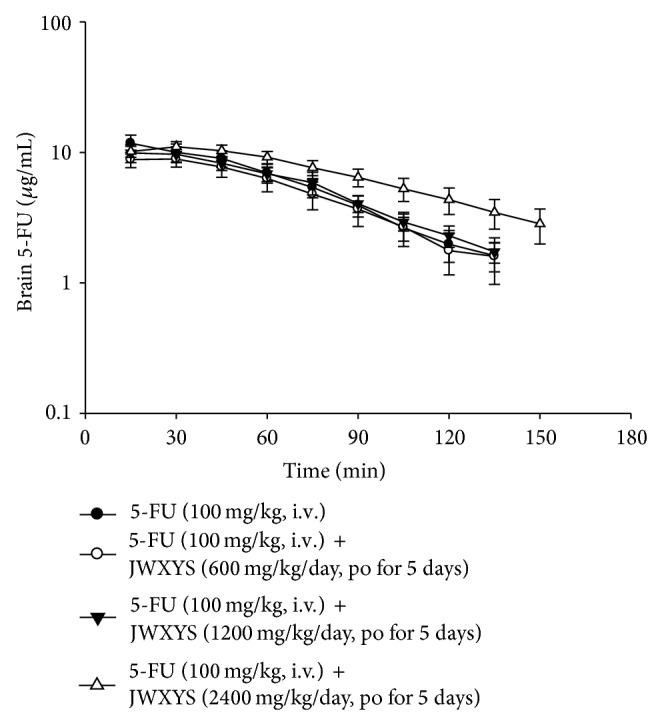
Concentration-time curve of 5-FU in rat brain dialysate after 5-FU administration (100 mg/kg, iv) alone and pretreated with a different dose of JWXYS (600, 1200, or 2400 mg/kg/day for 5 consecutive days). Data were expressed as means ± S.E.M. (*n* = 6).

Figure 6 shows the concentration-time curve for 5-FU in rat brain, which shows no difference between the 5-FU alone and daily and double dose of JWXYS pretreatment groups. The same phenomenon is observed in blood and the brain, where pretreatment with a high dose (2400 mg/kg/day) of JWXYS prolongs the residence time of 5-FU in the brain. In this study, 5-FU in the brain could not be detected within 150 min after 5-FU administration in the 5-FU alone and daily and double dose of JWXYS pretreatment groups, but it is observed in the high dose of JWXYS pretreatment group. The pharmacokinetic parameters of 5-FU in the brain are shown in Table 4. A daily or double dose of JWXYS has no significant effect on the AUC, *C*_max⁡_, *t*_1/2_, CL, or Vd. There is also no significant difference in AUC, *C*_max⁡_, and Vd between the 5-FU alone (894 ± 141 min *μ*g/mL, 11.8 ± 1.8 *μ*g/mL, and 6351 ± 953 mL/kg) and the high dose of JWXYS pretreatment group (1114 ± 128 min *μ*g/mL, 11.2 ± 1.1 *μ*g/mL, and 6525 ± 819 mL/kg). However, although there is no significant difference, AUC in the brain increases when 5-FU is coadministered with a high dose of JWXYS. The *t*_1/2_ value also increases significantly, from 31.9 ± 3.6 min to 47.2 ± 7.3 min, and the CL value decreases significantly, from 120 ± 23 mL/min per kg to 82.9 ± 11.0 mL/min per kg, when 5-FU is coadministered with a high dose of JWXYS.

It has been demonstrated that 5-FU can pass through the blood–brain barrier (BBB) by passive diffusion and affect the function of the central nervous system [3, 7], so it is important to determine whether the concentration of 5-FU changes with JWXYS coadministration. In this study, the ratio of 5-FU penetration is used to determine the variation in the concentration of 5-FU in the brain. The ratio of 5-FU penetration shows that JWXYS does not significantly influence the concentration of 5-FU across the BBB to the brain, but the penetrating ratio for 5-FU alone group is less than that for the high dose of JWXYS (2400 mg/kg/day) pretreatment group (Table 4).

### 3.7. Herbal-Drug Interaction

These results show that the herbal formula, JWXYS, in a daily or a double dose has no significant effect on the pharmacokinetics of 5-FU in blood and the brain. However, a high dose of JWXYS (2400 mg/kg/day) prolongs the residence time for 5-FU and causes the accumulation of the drug in blood and the brain and could increase the penetrating ratio for 5-FU through the BBB to the brain which could lead to significant damage to the brain. A previous study showed that the numerous metabolisms of drugs are related to cytochrome P450 (CYP). The CYP superfamily of monooxygenases is the main metabolic enzyme in human and is known to be expressed not only in the liver, but also in the intestine [26]. It has also been shown that CYP 1A is involved in the metabolism of 5-FU in rats. A study showed that CL of 5-FU is significantly faster when the expression of CYP 1A2 is increased [27]. Many studies have also shown that there are some components in herbal extract that inhibit the activity of the CYP superfamily, including CYP 1A2 [28–30]. A possible explanation is that a high dose of JWXYS supplies suppressant in a sufficient concentration to inhibit the expression of CYP 1A2 and leads to a reduction in the metabolic efficiency of 5-FU. This may be the reason why the *t*_1/2_ value in blood increases for the high dose of JWXYS pretreatment group.

A previous study also showed that the function of P-glycoprotein (P-gp) is to act as an efflux pump to block the accumulation of therapeutic drugs in brain tissue, so P-gp can discharge drugs, such as 5-FU, from brain [31]. Other studies have also shown that there are some components in herbs, such as flavonoids and 18-*β*-glycyrrhetic acid, which inhibit P-gp [32–34] that is contained in JWXYS. Therefore, an excessive dose of JWXYS might provide sufficient concentrations of flavonoids, 18-*β*-glycyrrhetic acid, or other suppressants to inhibit P-gp from pumping 5-FU out of the brain and lead to an increase in *t*_1/2_ and a reduction in CL, which could lead to an accumulation of the drug in the brain. However, P-gp not only exists in the BBB, but also is overexpressed in cancer cells. P-gp is a key factor in conferring the multidrug resistance (MDR) phenotype to cancer cells [35]. The function of P-gp is to remove drugs from cells, so overexpression of P-gp in cancer cells accelerates exclusion of the drug and causes MDR [36]. It has been reported that some herbal medicine extracts reverse the P-glycoprotein-mediated multidrug resistance of cells by inhibiting P-gp expression [37, 38]. JWXYS was prepared by mixing 10 crude herbs and the main components in extracted herbs were complicated. The study of bioactive constituents extracted by JWXYS is not clarified thoroughly. A previous study reveals that saikosaponin A and saikosaponin D in* Radix Bupleuri* (Chai-Hu), ferulic acid in* Radix Angelicae Sinensis* (Dang-Gui), and paeoniflorin in* Radix Paeoniae Alba* (Bai-Shao) and* Cortex Moutan Radicis* (Mu-Dan-Pi) would be extracted in JWXYS [39]. Furthermore, flavonoids in several herbs and glycyrrhizic acid in* Radix Glycyrrhizae Uralensis* (Zhi-Gan-Cao) are water-soluble components in JWXYS and could be extracted by water. And glycyrrhizic acid is hydrolyzed to 18-*β*-glycyrrhetic acid by intestinal bacteria after oral ingestion [40]. Combining the result of this study with previous ones, it is supposed that an excessive dose of JWXYS provides a sufficient concentration of flavonoids, 18-*β*-glycyrrhetic acid, or other active compounds to inhibit P-gp and to increase *t*_1/2_ and decrease CL in the brain. This might also affect the function of P-gp in cancer cells in reducing the probability of drug exclusion. This study demonstrates a potential treatment that improves the curative effect of 5-FU in inhibiting P-gp expression to reduce the chance of MDR in tumor cells by concomitant administration with JWXYS. It is also demonstrated that a daily or a double dose of JWXYS has no significant effect on the pharmacokinetics of 5-FU in rat blood and brain.

## 4. Conclusion

Currently, an increasing number of cancer patients use traditional Chinese medicine (TCM) and herbal medicine to reduce the side effects of other cancer therapies and to build up their strength. The interaction between the drug and the herbal medicine is extremely important. It has been proven that 5-FU can cause destruction in the brain. In this study, a continuous sampling device, microdialysis, is used to simultaneously monitor 5-FU in rat blood and in the brain. This study demonstrates that the herbal formulation, JWXYS, has no significant effect on the pharmacokinetics of 5-FU in the blood and the brain for a daily dosage regimen. Therefore, the concomitant administration of 5-FU with JWXYS is a feasible medication if there is a daily dose of JWXYS. However, caution should be exercised when prescribing an excessive dosage of JWXYS in a clinical application. A detailed clinical trial is required, to verify the herb-drug interaction for 5-FU with an excessive dosage of JWXYS.

## Figures and Tables

**Table 1:** Interday accuracy and precision for analysis of 5-FU in blood and brain microdialysate.

Nominal conc. (*µ*g/mL)	Observed conc. (*µ*g/mL)	Precision (%)	Accuracy (%)
Blood			
0.5	0.54 ± 0.04	7.74	7.06
1	1.03 ± 0.06	6.22	2.92
5	5.13 ± 0.13	2.46	2.61
10	9.75 ± 0.16	1.62	−2.49
50	50.07 ± 0.38	0.77	0.15
100	99.98 ± 0.18	0.18	−0.02
Brain			
0.1	0.10 ± 0.01	11.24	−3.52
0.25	0.23 ± 0.01	2.17	−6.85
0.5	0.49 ± 0.03	5.47	−1.52
1	1.03 ± 0.05	5.08	2.65
5	5.01 ± 0.11	2.22	0.15
10	9.99 ± 0.05	0.51	−0.06

Data were expressed as means ± S.D. (*n* = 6).

**Table 2:** Intraday accuracy and precision for analysis of 5-FU in blood and brain microdialysate.

Nominal conc. (*µ*g/mL)	Observed conc. (*µ*g/mL)	Precision (%)	Accuracy (%)
Blood			
0.5	0.55 ± 0.09	16.72	9.76
1	1.05 ± 0.10	9.29	5.45
5	5.05 ± 0.31	6.19	1.05
10	10.16 ± 0.38	3.75	1.56
50	49.42 ± 0.84	1.70	−1.16
100	100.27 ± 0.34	0.34	0.27
Brain			
0.1	0.10 ± 0.01	11.45	4.94
0.25	0.24 ± 0.01	3.98	−3.53
0.5	0.51 ± 0.02	4.55	1.44
1	1.06 ± 0.01	1.09	6.26
5	4.88 ± 0.06	1.21	−2.30
10	10.05 ± 0.03	0.27	0.49

Data were expressed as means ± S.D. (*n* = 6).

**Table 3:** *In vivo* microdialysis recovery (%) of 5-FU.

Concentration (*μ*g/mL)	Recovery (%)
Blood	
1	52.14 ± 2.45
10	49.14 ± 3.35
100	49.78 ± 2.88
Average	**50.35 ± 2.93**
Brain	
0.25	8.28 ± 0.81
1	9.12 ± 0.84
10	10.55 ± 1.30
Average	**9.32 ± 1.32**

Data were expressed as means ± S.D. (*n* = 3).

**Table 4:** Pharmacokinetic parameters of 5-FU in blood and the brain microdialysates after 5-FU administration (100 mg/kg, iv) alone and pretreated with a different dose of JWXYS (600, 1200, or 2400 mg/kg/day for 5 consecutive days) (*n* = 6).

Parameter	5-FU	5-FU + JWXYS (600 mg/kg/day, p.o.)	5-FU + JWXYS (1200 mg/kg/day, p.o.)	5-FU + JWXYS (2400 mg/kg/day, p.o.)
Blood				
AUC (min *μ*g/mL)	5020 ± 443	5516 ± 486	4801 ± 244	6159 ± 898
*C*_max⁡_ (*μ*g/mL)	107 ± 10	111 ± 9	98 ± 6	107 ± 8
*t*_1/2_ (min)	11.0 ± 0.6	13.1 ± 1.6	13.3 ± 1.6	25.6 ± 5.2^*^
CL (mL/min per kg)	20.8 ± 2.0	18.7 ± 1.4	21.0 ± 1.0	17.6 ± 2.6
Vd (mL/kg)	448 ± 51	406 ± 45	495 ± 76	575 ± 41^*^
Brain				
AUC (min *μ*g/mL)	894 ± 141	744 ± 133	834 ± 126	1114 ± 128
*C*_max⁡_ (*μ*g/mL)	11.8 ± 1.8	9.3 ± 1.2	10.1 ± 1.6	11.2 ± 1.1
*t*_1/2_ (min)	31.9 ± 3.6	28.9 ± 3.9	33.1 ± 2.9	47.2 ± 7.3^*^
CL (mL/min per kg)	120 ± 23	147 ± 28	122 ± 17.5	82.9 ± 11.0^*^
Vd (mL/kg)	6351 ± 953	7581 ± 1080	7114 ± 827	6525 ± 819
Ratio of penetration (%)	17.8 ± 2.8	14.6 ± 2.6	16.4 ± 2.5	24.2 ± 2.9

Data are expressed as mean ± S.E.M.

^*^ *P* < 0.05 comparing with that in 5-FU alone group.

The ratio of penetration (%) was calculated as follows: (AUC_brain_/AUC_blood_) × 100.

AUC_brain _meant AUC of brain in each group and AUC_blood _meant AUC of blood in 5-FU alone group.
